# Immobilized Lipase in Resolution of Ketoprofen Enantiomers: Examination of Biocatalysts Properties and Process Characterization

**DOI:** 10.3390/pharmaceutics14071443

**Published:** 2022-07-11

**Authors:** Oliwia Degórska, Daria Szada, Agata Zdarta, Wojciech Smułek, Teofil Jesionowski, Jakub Zdarta

**Affiliations:** Institute of Chemical Technology and Engineering, Faculty of Chemical Technology, Poznan University of Technology, Berdychowo 4, 60965 Poznan, Poland; dariaszada@gmail.com (D.S.); agata.zdarta@put.poznan.pl (A.Z.); wojciech.smulek@put.poznan.pl (W.S.); teofil.jesionowski@put.poznan.pl (T.J.)

**Keywords:** biocatalysis, ketoprofen, lipase, enzyme immobilization, active pharmaceutical ingredients

## Abstract

In this study, lipase from *Aspergillus niger* immobilized by physical immobilization by the adsorption interactions and partially interfacial activation and mixed physical immobilization via interfacial activation and ion exchange was used in the kinetic resolution of the ketoprofen racemic mixture. The FTIR spectra of samples after immobilization of enzyme-characteristic signals can be seen, and an increase in particle size diameters upon immobilization is observed, indicating efficient immobilization. The immobilization yield was on the level of 93% and 86% for immobilization unmodified and modified support, respectively, whereas activity recovery reached around 90% for both systems. The highest activity of immobilized biocatalysts was observed at pH 7 and temperature 40 °C and pH 8 and 20 °C for lipase immobilized by physical immobilization by the adsorption interactions and partially interfacial activation and mixed physical immobilization via interfacial activation and ion exchange, respectively. It was also shown that over a wide range of pH (from 7 to 10) and temperature (from 20 to 60 °C) both immobilized lipases retained over 80% of their relative activity, indicating improvement of enzyme stability. The best solvent during kinetic resolution of enantiomers was found to be phosphate buffer at pH 7, which obtained the highest efficiency of racemic ketoprofen methyl ester resolution at the level of over 51%, followed by enantiomeric excess 99.85% in the presence of biocatalyst obtained by physical immobilization by the adsorption interactions and partially interfacial activation.

## 1. Introduction

A significant number of active pharmaceutical ingredients (APIs) are chiral molecules. It often happens that different enantiomeric forms of a given compound exhibit various pharmacological properties and effects. A common reason for the use of racemic mixtures is mostly the cost of separation of the enantiomers, which exceeds the advantages of a possible increase in the activity [1]. There are substances where one of the enantiomers exerts a healing effect and the second one is toxic to the body [2]. The turning point of increasing the amount of toxicity tests before releasing the drug on the market was after a well-known tragedy that occurred in the late 1950s and early 1960s, where thalidomide was used for treatment of nausea during pregnancy. Use of this medication by pregnant women led to the deformation of the fetus, causing, among other effects, malformation of the correct limbs, heart disease, and malformations of inner and outer ear, due to the teratogenic liability of the drug. This resulted in the aggravation of toxicity tests and increased the systematicity of testing pharmaceutical active substances before they are approved for sale [3].

One of the groups of API containing drugs composed of chiral substances are non-steroidal anti-inflammatory drugs (NSAIDs) [4]. NSAIDs are the most commonly used group of drugs for inflammation and pain relief. One of them is ketoprofen (2-(3-benzoylphenyl)-propionic acid), which has an analgesic and anti-inflammatory effect and is still sold as a racemic mixture, even though both enantiomers show different activities. Enantiomer (S)- shows an anti-inflammatory effect, whereas (R)- enantiomer can prevent periodontal disease but also is analgesic and antipyretic [5,6].

Due to the increase in the importance of the synthesis of API in enantiomerically pure form, new ways of asymmetric synthesis are being sought [7]. At present, considering the published scientific papers, biocatalysis is a promising technique that allows the obtainment of enantiomerically pure products. On an industrial scale, enzymes such as *Candida antarctica* lipase type B can be used in the synthesis of chiral alcohols or amines [8]. This is dictated primarily by the pharmacological profile of individual forms of therapeutic compounds, as the main aim is to achieve the most favorable bioavailability while reducing side effects. Enzymes, especially hydrolases, can be successfully used during the synthesis of APIs, due to their properties, because stereo-, regio-, and chemoselectivity of the biocatalysts play the key role in this type of synthesis [7,9]. Additionally, enzymes are also non-toxic and fit into the assumptions of green chemistry, due to the lack of toxic solvents and mild process conditions [10]. Among other biocatalysts, hydrolases are readily used in asymmetric synthesis, mainly due to their availability and relatively low costs of their production. It is also important that the hydrolases do not require the presence of a cofactor to maintain high activity [11]. Among hydrolases, lipases are enzymes that are of particular importance because they show activity both in water and in organic solvents due to a feature characteristic of lipases, called interfacial activation. For lipases, the change of lid conformation during inhibition of helical lid, which rolls back from the active side, exposes a large hydrophobic surface, expanding the surface around active side [12]. This non polar surface is stabilized by the lipid environment, which is called interfacial activation. The interfacial activation occurs in the hydrophobic medium. Due to the presence of interfacial activation, lipases show high efficiency in the hydrolysis of particles containing carboxylic ester groups and which are aggregated in water [13,14]. Furthermore, lipases can not only carry out hydrolysis reactions in aqueous solvents but are also able to catalyze reactions such as acylation or esterification in nonaqueous conditions [15,16]. Due to these features, lipases are often used to obtain enantiomerically pure API [17,18]. The lipases most widely used for esterification of NSAIDs are lipases from *Candida rugosa* and *Rhizomucor miehei*, which catalyze the esterification of, for example, S-ibuprofen [19,20]. By contrast, lipase B from *Candida antarctica*, which catalyzes the reaction with a preference for (R)-enantiomer can be also used for the production of (S)-enantiomer, because it allows the direct synthesis of the (S)-enantiomer as an unreacted substrate of the enzymatic esterification [21]. Piacentini et al. used a multiphase reactor with lipase from *Candida rugosa* for kinetic resolution of (S,R)-naproxen methyl ester to obtain (S)-naproxen acid. The membrane emulsification technology was used for the production of a microstructured emulsion bioreactor using lipase as a catalyst and as a surfactant simultaneously. Enzyme loaded at the stable emulsion interface showed 100% of enantioselectivity and conversion rate, whereas during the classic stirring method the conversion was lower, reaching around 40–60%, and the enantiomeric excess fluctuated around 75% to 96% [22]. 

Despite having many advantages, enzymes are susceptible to the influence of the external environment, which can significantly interfere with their activity. Depending on the type of the enzyme, extreme operational conditions, which differ significantly from the optimal one, may change the biocatalyst structure, leading to its inhibition or inactivation. Hence, the immobilization process is widely used to stabilize the enzyme particle and extend the spectrum of its activity under various conditions [23]. Thanks to immobilization, the enzyme can also be removed more easily from the reaction medium, making it possible to skip the process of purifying the product from catalyst residues and enhancing the reusability of the catalyst [24]. Due to the fact that different immobilization techniques can be distinguished, the immobilization process should be chosen carefully to directly meet the process needs, because random immobilization processes can even cause a decrease of enzyme activity. Moreover, during immobilization, proper process conditions should be selected, such as pH, temperature, solvent, inhibitors, or different protein protectors, as these factors also influence enzymatic activity and stability [25]. Immobilization improves enzyme stabilization and limits diffusional limitations in different reaction media. In organic medium, insoluble enzymes tend to agglomerate and form clusters, whereas, due to the immobilization process, enzymes that are dispersed on the support surface are unable to agglomerate, creating active biocatalyst. Enzymes can be purified due to the immobilization process [26,27]. Additionally, proper immobilization process can tune enzyme selectivity, specificity, and activity, due to the conformational changes taking place in the enzyme molecule [28]. Finally, immobilization results in significant enzyme stabilization, especially when the enzyme is multipointly immobilized, as well as when a favorable environment for the enzyme is generated upon immobilization [29].

A very important part of preparing the immobilization process is choosing the right support. The use of nanoparticles has many advantages but it also has some boundaries. Nanoparticles have high specific surface area, nanometric particle size, and a large amount of space for the enzyme to attach. Nanoparticles tend to agglomerate and are hard to use on a large scale, so they need to be modified with, for example, magnetite to separate them easily from the reaction medium. Immobilization also depends on the porosity of the material. If the material is porous, pores can be contaminated with solid particles and the enzyme immobilized inside the pores will be unable to meet the substrates during reaction, creating biocatalyst of low activity. In choosing the support it is important to pay attention to obtaining a suitable enzyme orientation and high activity [30]. It should also be added that immobilization can be successfully performed on nanoparticles via adsorption (ionic exchange, hydrophobic adsorption, and affinity adsorption), covalent binding (via covalent attachment, anionic exchange, and interfacial activation), or entrapment, improving enzymes stability, its selectivity, and simplicity of mixture purifying after reaction [31]. Additionally immobilized enzymes usually show greater thermal stability than the native form, due to the protective effect of the support [32]. For instance, Foresti et al. used the commercial product Novozym 435 (*Candida antarctica* lipase B immobilized on the acrylic resin) and Lipozyme RM IM (*Rhizomucor miehei* lipase immobilized on a macroporous ion-exchange resin) for the synthesis of S-ibuprofen and obtained an enantiomeric excess of 54% and 63%, respectively. They investigated the addition of water to the ethanol during the reaction and the most suitable water content, which was a compromise between good efficiency and enantiomeric excess, was found to be 4.8% *v*/*v* [33]. Further, Wang et al. obtained (S)-citalopram, which is a selective serotonin reuptake inhibitor for treating depression, anxiety, and panic disorder. During the chemoenzymatic reaction, supported by Novozym 435, they conducted the kinetic and cyclic resolution of racemic diol. An enantiomeric excess of 98% was obtained and the efficiency of the reaction reached 97.8% [34].

**Scheme 1:**
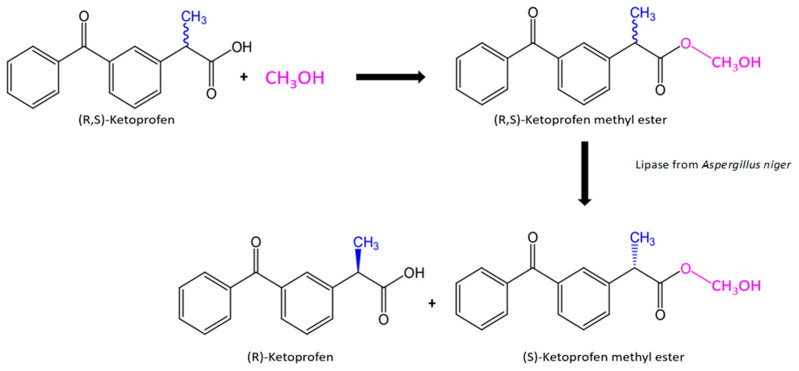
Reaction path of chemical synthesis and enzymatic enantioselective resolution of ketoprofen methyl ester.

In this study, a novel attempt has been made concerning the use lipase from *Aspergillus niger* immobilized onto silica surface in the synthesis of enantiomerically pure (R)-ketoprofen in aqueous medium from a racemic mixture of ketoprofen. The most suitable process parameters including temperature or pH for the highest enzyme activity have been examined, as well as the effect of storage time, thermal stability, and reusability. To verify the possible practical application of the produced biosystems, tests were performed in buffer solution and organic solvents using various amounts of biocatalyst to examine the efficiency of kinetic resolution of the ketoprofen racemic mixture. The obtained results of the enantiomeric excess and the efficiency of the process indicate the high application potential of the biocatalysts produced during the separation reaction of the racemic mixture of ketoprofen. The reaction path conducted during this research is presented in Scheme 1 below. However, future research is still required to further increase the yield of the reaction and improvement of process control.

## 2. Materials and Methods

### 2.1. Chemicals and Materials 

Lipase from *Aspergillus niger*, racemic ketoprofen, para-nitrophenyl palmitate, *p*-nitrophenol (pNPP), (3-aminopropyl)triethoxysilane (APTES), Bradford reagent used for examination of the quantity of immobilized enzyme, N,O-Bis(trimethylsilyl)trifluoroacetamide (BSTFA), phosphate, acetate and TRIS buffers at various pH, and silica nanoparticles were acquired from Sigma-Aldrich (Saint Louis, MO, USA). Hexane, sodium carbonate, sodium hydrogencarbonate, and methanol were purchased from Chempur, whereas chloroform was acquired from POCH. 

### 2.2. Lipase Immobilization by Physical Immobilization by the Adsorption Interactions and Partially Interfacial Activation and Mixed Physical Immobilization via Interfacial Activation and Ion Exchange

The first stage of the research was lipase physical immobilization by the adsorption interactions and partially interfacial activation and mixed physical immobilization via interfacial activation and ion exchange. In the physical immobilization by the adsorption interactions and partially interfacial activation, the lipase enzyme was immobilized on the support without its modification. Immobilization was performed in a 100 mL flat bottom flask closed with parafilm. For immobilization, 2 g of silica was weighed into the flask, to which 45 mL of 5 mg/mL lipase solution in 100 mM phosphate buffer at pH 7 was added. The sealed system was then shaken at 30 °C, 200 rpm, for 1 h and 24 h in a shaking incubator (IKA KS 4000i Control, Warsaw, Poland). To further separate the carrier with immobilized lipase from the reaction medium, the sample was transferred to a conical tube and centrifuged for 15 min at 4000× *g* rpm in a centrifuge (Eppendorf Centrifuge 5810 R, Hamburg, Germany). The immobilized enzyme was separated from the remainder of the mixture and left to dry in the open air. The solutions obtained after centrifugation were kept for further analysis.

In the mixed physical immobilization via interfacial activation and ion exchange, prior to immobilization, the support was modified with APTES solution. For this reason, 0.6 g of APTES was diluted in 20 mL of the solution of methanol/water (4/1 *v*/*v*). Then, 2 g of the carrier was moistened with a sprayer that contained APTES solution. Afterwards the support was dried in an oven in temperature of 80 °C for 6 h. The next step was the immobilization process. For immobilization, 2 g of modified silica was weighed into the flask, to which 45 mL of 5 mg/mL lipase solution in 100 mM phosphate buffer at pH 7 was added. The sealed system was then shaken at 30 °C, 200 rpm, for 1 h and 24 h in a shaking incubator (IKA KS 4000i Control). After immobilization, samples were centrifuged for 15 min at 4000× *g* rpm in a centrifuge (Eppendorf Centrifuge 5810 R). The immobilized enzyme was separated from the remainder of the mixture and left to dry in the open air. The solutions obtained after centrifugation were kept for further analysis.

### 2.3. Effect of Reaction Parameters (pH, T), Storage Stability, Thermal Stability, and Reusability

In order to select the suitable process parameters for the immobilized lipase, the influence of temperature and pH on the activity of the produced biocatalytic systems prepared by immobilization by physical immobilization by the adsorption interactions and partially interfacial activation and mixed physical immobilization via interfacial activation and ion exchange was examined. For pH investigation, 2 mL of buffer at specific pH was added to Eppendorf tubes containing 50 mg of biocatalyst, followed by addition of 1 mL of para-nitrophenyl palmitate solution at the concentration of 15 mM and reacted for 5 min at pH values: 4, 5, 6, 7, 8, 9, and 10 in an Eppendorf ThermoMixer C (Hamburg, Germany). For the specific pH value different buffers were used: acetate buffer for pH 4 and 5, phosphate buffer for pH from 6 to 8, and TRIS buffer to reach pH 9 and 10. Reactions were conducted in the temperature of 30 °C.

For the hydrolysis of *p*-nitrophenyl palmitate at varying temperatures, 50 mg of the immobilized enzyme prepared by physical immobilization by the adsorption interactions and partially interfacial activation and mixed physical immobilization via interfacial activation and ion exchange methods was placed in Eppendorf tubes and the 2 mL of phosphate buffer at pH 7 was added to the tubes containing 50 mg of biocatalyst, followed by addition of 1 mL of 15 mM para-nitrophenyl palmitate solution, and reacted for 5 min at temperatures: 10, 20, 30, 40, 50, 60 °C. For both analyzed parameters the reactions were terminated with 1 mL of Na_2_CO_3_ solution at the concentration 0.5 M and centrifuged afterwards. 

The thermal stability was investigated to determine the activity of the biocatalysts prepared by physical immobilization by the adsorption interactions and partially interfacial activation and mixed physical immobilization via interfacial activation and ion exchange, under heat stress. Briefly, 50 mg of biocatalyst with 2 mL of buffer phosphate at pH 7 was incubated for 2 h in 6 different temperatures: 30, 40, 50, 60, 70, and 80 °C. Likewise in the previous steps, after this time to each Eppendorf tube 1 mL of 15 mM *p*-nitrophenyl palmitate was added and the reaction was conducted for 5 min. Afterwards the termination of reaction was performed with 1 mL of 0.5 M Na_2_CO_3_ solution and centrifuged.

For the reusability study, 10 reaction cycles were performed at the temperature of 30 °C. Then, 2 mL of phosphate buffer at pH 7, 1 mL of 15 mM para-nitrophenyl palmitate and 50 mg of biocatalyst was added to the Eppendorf tube and the reaction was conducted for 5 min in Eppendorf ThermoMixer C. After this time, samples were centrifuged in a centrifuge (Eppendorf Centrifuge 5810 R) for 15 min at 4000× *g* rpm and obtained precipitate was washed several times by phosphate buffer and was used in the next reaction. In these experiments, initial enzyme activity was defined as 100% activity. 

Storage stability of free lipase and biocatalysts after immobilization were examined based on above-described model reaction of para-nitrophenyl palmitate hydrolysis over 30 days of storage at 4 °C in phosphate buffer at pH 7. The relative activity was measured every day. In these experiments, initial enzyme activity was defined as 100% activity.

All of the experiments performed with immobilized enzymes were also performed using free lipase, for comparison. The solutions obtained after the processes were examined using UV-vis spectroscopy in order to check the efficiency of para-nitrophenyl palmitate conversion at particular temperatures and pH values as well as over study. The increase in absorbance was measured at 410 nm and is caused by the release of *p*-nitrophenol during enzymatic hydrolysis. A standard curve prepared based on solutions of *p*-nitrophenol of known concentration was used to determine the final concentration of the substrate/product in the solution. One unit of the enzyme activity was defined as the release of 1 mmol of pNP per one minute under the measurement conditions. In tests on effect of pH and temperature, the highest noticed activity was defined as 100% relative activity. 

### 2.4. Immobilization Yield, Amount of Immobilized Enzyme, and Specific Activity

The amount of the immobilized enzyme was calculated based on the spectrophotometric measurements using the Bradford method [35]. In a quartz cuvette, 4 mL of the Bradford reagent was mixed with 800 µL of the analyzed protein solution and 100 µL water, and the analysis was performed 10 min after the preparation of the mixture. UV/vis measurements were made at wavelength 595 nm, using a JASCO V650 spectrophotometer (Jasco, Tokyo, Japan). The amount of immobilized enzyme (mg/g) was determined as the difference between the initial amount of lipase and the final enzyme amount in the mixture after immobilization, relative to the mass of the silica nanoparticles. Immobilization yield (%) was calculated following Equation (1), whereas activity recovery was calculated using Equation (2):

(1) $$mmobilization\;yield\;\left(\%\right)=\frac{A_{i}-A_{f}}{A_{i}}\times 100\%$$

(2) $$Activity\;recovery\;\left(\%\right)=\frac{A_{t}}{A_{i}}\times 100\%$$

where *A_i_* denotes the initial activity of lipase added to the immobilization medium, *A_f_* denotes the total activity of the enzyme in the supernatant and washing solution after the immobilization, and *A_t_* denotes the activity of the immobilized lipase.

To determine specific activity and relative activity of free and immobilized lipase, spectrophotometric measurements were performed, based on a model pNP reaction. The reaction was carried out for 5 min at 30 °C. Using the standard calibration curve for pNP, the specific activity of the free and immobilized enzyme (U/mg) was calculated as the initial enzyme activity retained per unit mass of enzyme and per unit mass of enzyme and support. The activity retention (%) of immobilized lipase is presented as the percentage activity of the immobilized lipase, relative to the catalytic activity of the free enzyme.

### 2.5. Synthesis of Racemic Ketoprofen Methyl Ester 

For racemic ketoprofen methyl ester production, 1 g of racemic ketoprofen was diluted in the mixture of 30 mL of methanol and 300 mL of chloroform with addition of a few drops of sulfuric acid(VI) as catalyst of the reaction. The mixture was heated and refluxed in 60 °C for 3 h in the bathwater. The obtained product was washed three times alternately with 1 M NaHCO_3_ and distilled water. Afterwards, the obtained product was left to dry in ambient conditions and used in further reactions. 

### 2.6. Enzymatic Resolution of Racemic Ketoprofen Methyl Ester 

The hydrolysis reactions were performed in phosphate buffer at pH 7 but also in hexane and isopropyl alcohol in batch reaction system. The amount of 10.7 mg of ketoprofen methyl ester and 10 mg of free enzyme or amount of biocatalytic systems produced that contains 10 mg of immobilized lipase was placed in the Eppendorf tubes and 2 mL of the buffer or organic solvent was added. For comparison, 15 mg and 20 mg of immobilized lipase was tested. The processes were performed for 24 h and 96 h for each sample, regardless of the type of solvent or biocatalyst and its amount. To confirm the effective obtaining of ketoprofen enantiomers, racemic resolution and determination of the enantiomeric excess and the efficiency of the enzymatic conversion, gas chromatography coupled with mass-spectrometry (GC-MS) was used. Initially, the samples were concentrated to evaporate the solvent. Afterwards, the samples were derivatized by adding 300 µL of BSTFA and heated at 60 °C for 2 h. Subsequently, samples were subjected to GC-MS analysis (Pegasus 4D from Leco, Vouersweg, The Netherlands). Quantitative analysis was performed with a Capillary GC Column Cydex-B, GC Column 0.22 mm ID 0.25 µm Film 25 mL, Ea from Phenomenex. The analysis was performed with the oven temperature programmed as follows: 40 °C for one minute, increased to 120 °C with a rate 5 °C/min, then 120 °C for 50 min.

### 2.7. Physicochemical Characterisation

The particle size distribution was obtained on a Zetasizer Nano ZS instrument (Malvern Instruments Ltd., Malvern, UK). The analyzer measures particle sizes in the range 0.6–6000 nm and operates based on the non-invasive backscattering (NIBS) technique. For sample preparation, the material was dispersed in isopropanol in a Sonic-3 ultrasonic bath (Polsonic, Warsaw, Poland) for 15 min.

The FTIR spectroscopy was used to identify functional groups present in the resulting systems. The obtained samples were analyzed by FTIR-ATR technique. The ATR (Attenuated Total Reflectance) technique uses the phenomenon of weakened total infrared radiation. The adapter is equipped with a diamond, which is responsible for the multiple reflection of infrared radiation. The analysis of the obtained materials was carried out using the Vertex 70 device from Bruker with a resolution of 0.4 cm^−1^.

The effect of Triton X-100 1%, NaCl solutions at different concentrations (0.1 and 0.5 M) and mixture of TX-100 1% + NaCl 0.1 M on the relative activity and amount of immobilized lipase from *Aspergillus niger* was evaluated for 6 h. During this experiment, the immobilized enzyme was dispersed in sodium chloride, Triton X-100 or mixture solution. After each hour, the relative activity of the immobilized enzyme was evaluated based on the hydrolysis reaction of the enzyme substrate, whereas the amount of immobilized lipase retained on the support was determined using Bradford method.

### 2.8. Efficiency and Enantiomeric Excess of the Kinetic Resolution Process of the Racemic Mixture

In order to determine the efficiency of the enzymatic conversion of the ketoprofen methyl ester racemate to (S)- and (R)-ketoprofen, Equation (3) was used:

(3) $$W=\frac{Cp}{Cpr}\times 100\%$$

where 𝑊 denotes efficiency of the enzymatic conversion process of the racemic mixture of ketoprofen methyl ester, 𝐶𝑝 is concentration of products obtained in the sample after enzymatic conversion (mg/mL), and 𝐶𝑝𝑟 is concentration of products obtained as a result of enzymatic conversion resulting from the stoichiometry of the reaction (mg/mL).

To determine the enantiomeric excess (*ee*, %) of (R)-ketoprofen over (S)-ketoprofen, Equation (4) was used:

(4) $$ee=\frac{R-S}{R+S}\times 100\%$$

where *S* represents concentration of enantiomer (S)-ketoprofen (mg/mL) and *R* denotes concentration of enantiomer (R)-ketoprofen (mg/mL).

## 3. Results and Discussion

### 3.1. Characterisation of the Produced Biocatalytic Systems

The process performed as well as the resulting biocatalytic systems were characterized in terms of the process yield and the activity of the immobilized enzymes. From Table 1 it can be seen that after 24 h of lipase physical immobilization by adsorption interactions and partially interfacial activation (immobilization with nonmodified support) and mixed physical immobilization via interfacial activation and ion exchange (immobilization with modified support), relatively high immobilization yields of 93% and 86%, respectively, were achieved, resulting in a high amount of immobilized lipase of 205 mg/g and 187 mg/g. These data suggest that immobilization on nonmodified support could be partially multilayered which explains the higher amount of immobilized lipase. It is also evident from the data that 90% and 87% activity was retained by the lipase immobilized on nonmodified and modified support respectively, as its specific activity was 53 and 49 U/mg, compared with 57 U/mg for the free enzyme. Lower activity retention for the mixed physical immobilization via interfacial activation and ion exchange may be related to the interference in the structure of the enzyme and changes in enzyme structure upon immobilization related to the formation of chemical bonds between the enzyme and the carrier, which could reduce its activity and partially block the enzyme active sites due to spontaneous and uncontrolled immobilization [36].

These results prove the successful immobilization of the enzyme and suggest the high activity of the produced biocatalysts. In another study, it was reported that lipase from *Rhizopus oryzae* immobilized on the silica aerogel by adsorption results in 95% of immobilization yield, mainly due to the presence of numerous hydroxyl groups on the aerogel surface [37].

**Figure 1:**
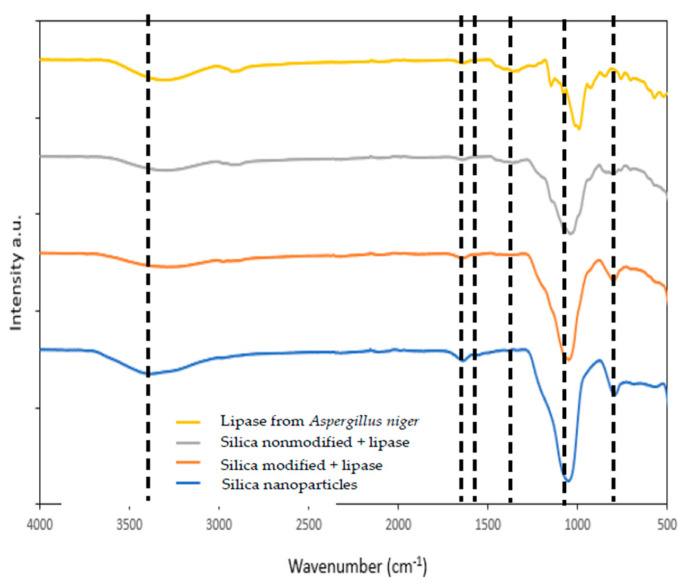
FTIR spectra of nanosilica (blue line), lipase from *Aspergillus niger* (yellow line), and nanosilica with immobilized lipase by physical immobilization by the adsorption interactions and partially interfacial activation and mixed physical immobilization via interfacial activation and ion exchange.

Particle size distribution is an analysis that enables the changes in the support properties upon immobilization to be followed (Table 2). The obtained data show that the pure silica nanoparticles are in the size range typical for nanomaterials, from 59 to 220 nm and the PdI value is low (0.225), which indicates the uniformity of the support. For the samples after lipase physical immobilization by adsorption interactions and partially interfacial activation, particle size ranges from 91 to 1106 nm with maximum volume contributions of 31.4% and 25.4% coming from diameters of 164 nm and 141 nm, respectively. Particle sizes from 1484 nm to 6439 nm were noticed for silica with lipase immobilized by mixed physical immobilization via interfacial activation and ion exchange, whereas the maximum volume contributions of 18% and 18.5% for particles at diameters of 3580 and 4145 nm were noticed. A significant increase in the particle size diameters after immobilization indicates that the immobilization processes were conducted successfully and lipase molecules were deposited onto the silica surface. Further, this increase might be due to the fact that enzyme particles are bigger and may create clusters with silica support [38]. Nevertheless, significantly higher particle size after mixed physical immobilization via interfacial activation and ion exchange indicates that the presence of surface modifier affects the size of the formed biocatalyst. To additionally confirm the effective immobilization of the lipase from *Aspergillus niger* and to characterize the individual functional groups in the analyzed materials, FTIR spectroscopy was used (Figure 1).

Figure 1 shows the FTIR spectra with bands characteristic of vibrations of different functional moieties assigned to the silica and lipase functional groups (grey and red lines). In the FTIR spectrum of SiO_2_, in the wavenumber range 900–500 cm^−1^, signals characteristic of the asymmetric and symmetric bending and stretching vibrations of Si–O groups are visible. At 1100 cm^−1^ band of the stretching, vibrations of Si–O–Si groups occur, whereas at a wavenumber of around 1630 cm^−1^ the signal assigned to the stretching vibrations of H_2_O occurs, indicating the presence of physically adsorbed water. At a wavelength of approx. 3400 cm^−1^, a wide band of stretching vibrations of hydroxyl groups occurs, proving the presence of these groups on the silica surface and suggesting that mainly these moieties are responsible for enzyme binding.

The FTIR spectrum of free lipase (yellow line) below 1000 cm^−1^ shows a few signals assigned to the stretching and bending vibrations of C–C bonds that form the enzyme skeleton [39]. The signal of stretching vibrations of C–O bonds appears in the spectrum at 1020 cm^−1^. The signals at 1250 cm^−1^, 1545 cm^−1^, and 1650 cm^−1^ are characteristic of the stretching vibrations of amide I, amide II, and amide III bands, respectively. The band at around 2900 cm^−1^ is assigned to the stretching C–H vibrations. Finally, a band between 3600 and 3200 cm^−1^ also occurs, which might be assigned to the stretching vibrations of –OH and –NH groups. Analysis of the FTIR spectrum of the lipase confirmed the presence of a number of functional groups in the enzyme structure, which can be employed for binding the enzyme to the support.

Both spectra of silica with immobilized lipase show signals from silica and enzyme, which confirms successful enzyme immobilization. The most important are signals generated by the amide I, II, and III bands observed in FTIR spectra upon immobilization at 1640 cm^−1^, 1540 cm^−1^, and 1250 cm^−1^. A slight shift in wavenumber maxima of these signals (of around 10 cm^−1^), as compared to the free enzyme, indicates successful immobilization and changes in the chemical environment of the enzyme after its deposition onto the silica surface and suggests the creation of interactions between nanosilica and the enzyme [40,41]. These changes are more visible for the silica with lipase immobilized by mixed physical immobilization via interfacial activation and ion exchange, because this type of immobilization tends to create more changes in the enzyme structure, due to the formation of chemical bonds between enzyme and support.

**Figure 2:**
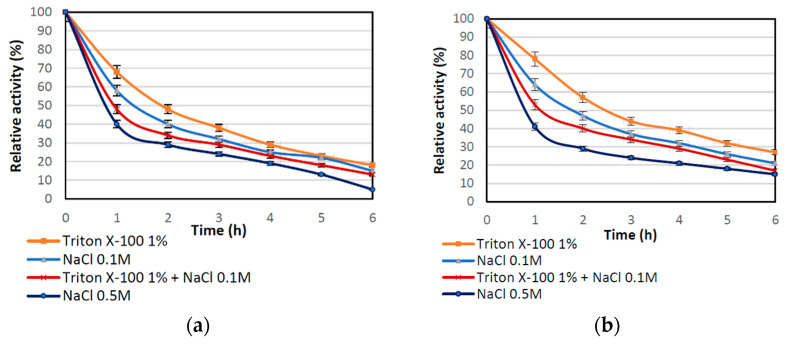
Effect of Triton X-100 and NaCl solution on the relative activity of immobilized enzyme: (**a**) by the adsorption interactions and partially interfacial activation (**b**) by mixed physical immobilization via interfacial activation and ion exchange. All experiments were performed in duplicate and the results presented in each of the graphs are shown as a mean value, for which standard deviation (error value) does not exceed 5%.

To verify type of enzyme-support interaction, elution tests were performed using various eluents. Figure 2 shows the correlation that the relative activity of the immobilized lipase treated with 1% Triton X-100 (TX-100), NaCl solutions at different molar concentrations, and combination of TX-100 and NaCl declined gradually during the 6 h treatment, which suggests elution of the enzyme from the matrix. However, partial inactivation of the immobilized enzyme by the eluent could not be excluded as both compounds might negatively affect lipase activity. The lowest drop of relative activity, for both biocatalysts produced, were obtained for TX-100, indicating that surfactant has the lowest effect on enzyme elution. After incubation in NaCl solutions, a more significant drop of activity was observed. This might be due to the ion exchange that occurs between enzyme immobilized by ion exchange and sodium chloride ions. Higher relative activities occurring for immobilization with modified support may be related to the fact that introduction of amine groups in modification process leads to formation of stronger interactions between enzyme and the support and immobilization, not only via interfacial activation by also via ion exchange. Furthermore, in Table 3 the amount of immobilized enzyme that remained after 6 h of desorption tests on the surface of support is presented. These data show a gradual drop of the amount of immobilized lipase on both supports; however, a more pronounced decrease was noticed for samples obtained using unmodified support. These results correspond with the data on relative activity after desorption experiments. The highest amount of lipase retained on the support (around 52 mg/g) was obtained for modified support after leaching using TX-100, which corresponds to the relative activity of around 30%. By contrast, more than 75% of the enzyme (over 150 mg of the lipase from 1 g of the support) was eluted from nonmodified silica after 0.5 M NaCl treatment. The collected results clearly show that support modification provides more stable enzyme-support interactions, mainly via interfacial activation and ion exchange.

### 3.2. Parameters Affecting Enzymes Activity

The reaction environment is very important for enzymes to obtain the highest possible activity. Due to their nature, enzymes are sensitive to pH and temperature and these parameters stimulate the enzyme’s inhibition and inactivation. Taking into account the aforementioned dependencies, the temperature and pH were tested over a wide scale to select the most suitable process conditions for immobilized lipase.

**Figure 3:**
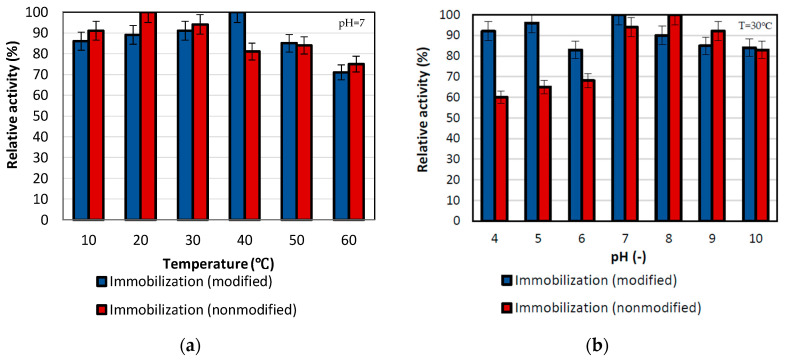
Effect of (**a**) temperature and (**b**) pH on immobilized lipase activity. All experiments were performed in duplicate and the results presented in each of the graphs are shown as a mean value, for which standard deviation (error value) does not exceed 5%. During investigation in varying temperature, reactions were performed in 100 mM phosphate buffer at pH 7, while reactions conducted in different pH were performed in the temperature of 30 °C.

The effect of temperature was determined during *p*-nitrophenyl palmitate conversion over a wide temperature range from 30 °C to 80 °C (Figure 3). The highest activity for the performed reaction was obtained for lipase immobilized by mixed physical immobilization via interfacial activation and ion exchange with maximum activity at 20 °C. By contrast, for the temperature of 40 °C, the maximum relative activity of the enzyme immobilized by the adsorption interactions and partially interfacial activation method was obtained. Additionally, high activities, of around 85% were noticed for both samples at the temperature of 50 °C. Nevertheless, over the temperature range from 10 °C to 50 °C, the activity of both types of biocatalysts exceeded 80%, which indicates considerable thermal stability of both immobilized lipases. This is related to the protective effect of the support on the enzyme structure [42]. Additionally, it is not possible to unequivocally determine which of the biocatalysts is the most stable in the analyzed temperature range. This is due to many dependencies, including the shift of the optimum temperature of the enzyme operation and changes in the enzyme structure. Nevertheless, taking into account the obtained results, both techniques are effective in improving the stability of the enzyme to temperature exposure. Immobilization is a way of improving enzymes properties; an examples is found in a study by Pinheiro et al. They immobilized lipase B from *Candida antarctica* on the chitosan-divinyl sulfone and also obtained high relative activity in a wide range of pH and temperature [43].

To determine the optimum pH, the tests were performed in the pH range of 4–10 using a 15 mM substrate solution in buffer at specific pH, at the temperature of 30 °C (Figure 3b). It can be seen that the highest activity of immobilized lipase is observed at pH 7 for enzyme deposited on the SiO_2_ by immobilization by the adsorption interactions and partially interfacial activation, whereas for mixed physical immobilization via interfacial activation and ion exchange the highest relative activity of the enzyme was noticed at pH 8. After that pH value, the relative activity for both biocatalysts slightly decreased, but the drop in activity was not greater than 20%. The shift of the pH maximum in the case of the sample obtained as a result of mixed physical immobilization via interfacial activation and ion exchange was associated with the formation of interactions between the carrier and the enzyme, which affects lipase structure and results in shifting of pH optima. Nevertheless, both of the biocatalysts obtained show high relative activity over a broad range of pH from 7 to 10, where the activity was at a level above 85%. This strictly indicates the improvement of the stability of the enzyme after immobilization at different pH, which is caused by rigidization of the enzyme structure upon immobilization [44].

For the native form of the enzyme (data not presented), even the smallest changes in process parameters determine a rapid drop in relative activity. The highest activity was observed at pH 7 and temperature 30 °C, which are optimum work parameters for lipase from *Aspergillus niger* [45]. When changing the process parameters, the activity of free lipase decreases sharply and is even lower than 30% at pH ranges from 4 to 5 and from 9 to 10. Similar values were obtained for the temperatures 10, 50, and 60 °C, where the relative activity fluctuated from 20 to 50%. Low relative activity is related to the enzyme’s sensitivity to external factors, which can significantly inhibit its activity or even irreversibly degrade its structure [46]. Zdarta et al. investigated immobilization processes of lipase B from *Candida antarctica* (CALB) on the 3D spongin-based scaffolds from *Hippospongia communis* marine demosponge. The obtained data also confirmed a significant improvement in the stability of the enzyme over a wide range of temperatures and pH. During the tests performed over a wide temperature range, the obtained biocatalysts retained over 80% of activity over temperature from 30 °C to 60 °C and pH from 6 to 9 [47].

**Figure 4:**
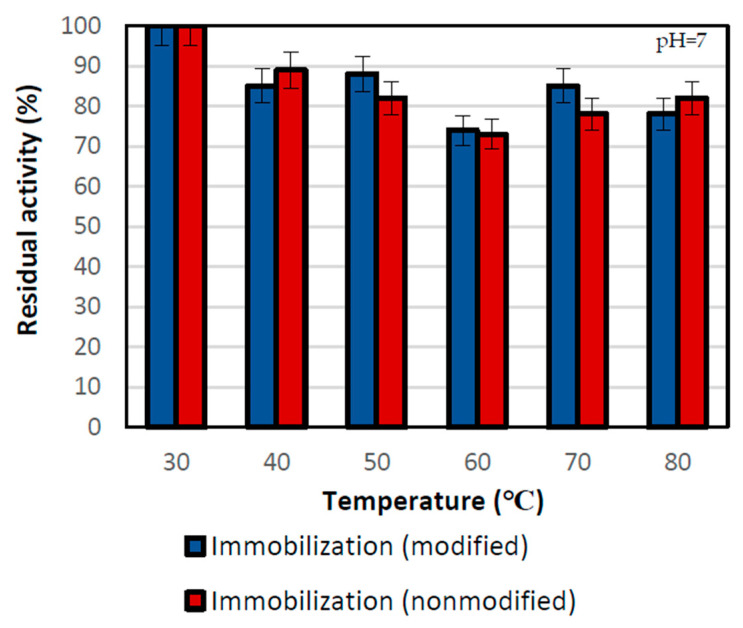
Thermal stability of lipase immobilized by two physical techniques: by the adsorption interactions and partially interfacial activation and mixed physical immobilization via interfacial activation and ion exchange. All experiments were performed in duplicate and the results presented in each of the graphs are presented as a mean value with standard deviation (error value). The thermal stability test was performed in the 100 mM phosphate buffer at pH 7 in different temperatures.

The thermal stability of the biocatalyst was also one of the key analyses in this study. It was examined after a 2 h incubation of immobilized lipases at the desired temperature ranging from 30 ℃ to 80 °C (Figure 4). 

The highest thermal stability of the biocatalyst was noticed at the lowest temperature of 30 °C, where both immobilized lipases showed the highest residual activity. Above that temperature, the residual activities for both samples varied but were lower as compared to 30 °C due to partial lipase inactivation. At the temperature of 50 °C, the relative activities were at the level of around 88% for the sample performed with nonmodified support and 81% for the modified support immobilization one. However, even at the temperature of 80 °C, both types of biocatalysts retained about 80% of their relative activity. At the high temperature, silica support acts as a heat absorber and provides a protective effect for immobilized enzymes. This results in significant thermal stability of biocatalyst supported by the stabilization of the enzyme structure, and its protection against denaturation and defragmentation [48]. 

**Figure 5:**
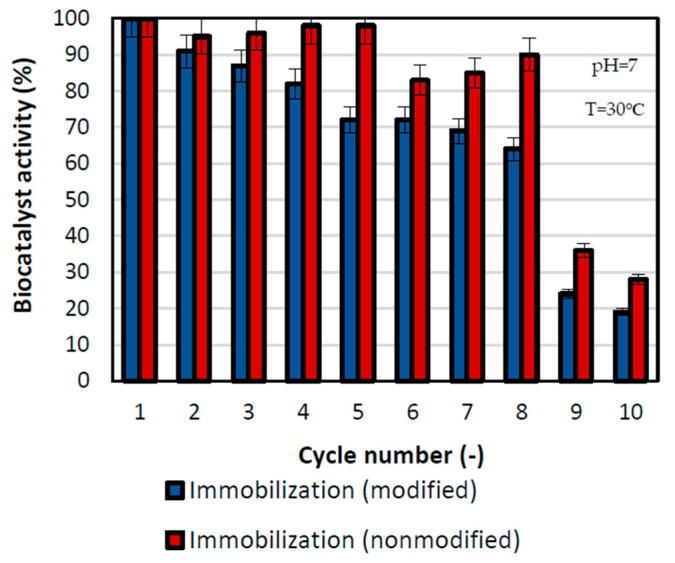
Reusability of the biocatalyst obtained by physical immobilization by the adsorption interactions and partially interfacial activation and mixed physical immobilization via interfacial activation and ion exchange. All experiments were performed in duplicate and the results presented in each of the graphs are presented as a mean value, for which standard deviation (error value) does not exceed 5%. The reusability tests were performed in the temperature of 30 °C and in phosphate buffer at pH 7.

One of the most crucial parameters for practical application of produced biocatalysts is the possibility of biocatalyst reuse, which is why during this research a series of replications of the reactions were performed (Figure 5). Reusability is an important aspect of enzymes used in a practical way that reduces the costs of their exploitation. After the first cycle, the biocatalyst’s activities were at the level of 100%, and after that, the activity slightly decreased. For the sample prepared by immobilization with modified support, the activity was higher, in all reaction cycles, than in the case of immobilization with nonmodified support. The rapid drop in activity was visible after the 9th cycle, where the relative activity of the biocatalyst prepared by the physical immobilization by adsorption interactions was at the level of 26% and the one by mixed physical immobilization was at the level of 38%. Nevertheless, over eight repeated cycles, more than 60% of relative activity was retained by the lipase immobilized on nonmodified support, whereas over 80% of activity was preserved by the biocatalyst made by the mixed physical immobilization method. The greater decrease in the activity of biocatalysts immobilized on nonmodified support may be related to the greater inhibition of the enzyme by the substrates and reaction products. Beyond inhibition, the observed decrease may also be related to enzyme deactivation and its partial washing out. During immobilization on modified support, the enzyme is strongly attached to the support’s surface by chemical bonds, whereas the enzyme immobilized on nonmodified support is attached with the use of weak hydrogen bonds and van der Waals forces, leading to faster enzyme elution. Nevertheless. preservation of high activity over the following catalytic cycles indicates a stable binding of the enzyme to the support. 

**Figure 6:**
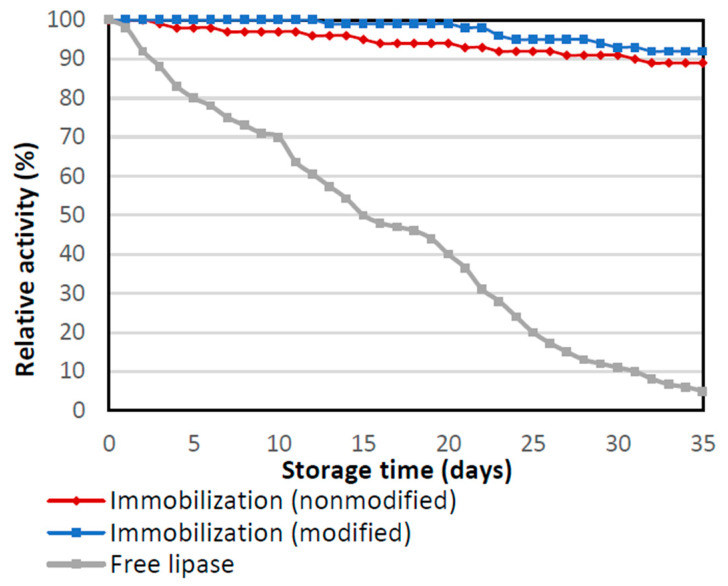
Storage stability of the native enzyme and lipase immobilized by physical immobilization by the adsorption interactions and partially interfacial activation (immobilization nonmodified) and mixed physical immobilization via interfacial activation and ion exchange (immobilization modified). All experiments were performed in duplicate and the results presented in each of the graphs are shown as a mean value, for which standard deviation (error value) does not exceed 5%.

Storage stability was also investigated for native and immobilized forms of lipase during performing of storage stability. Obtained biocatalysts were stored in PBS 7, at the temperature of 4 °C for 35 days (Figure 6). It is noticeable that for native enzyme relative activity decreased from the first day of storage and after 35 days showed less than 12% of its activity. On the other hand, for enzymes immobilized by physical immobilization by the adsorption interactions and partially interfacial activation and mixed physical immobilization via interfacial activation and ion exchange, the relative activity was very high even at the end of the storage test. After 35 days both immobilized biocatalysts remained highly active with relative activity above 90%. Slightly higher activity for lipase immobilized by mixed physical immobilization via interfacial activation and ion exchange is related to the higher stabilization of enzyme structure due to stronger chemical bonds and limited enzyme leaching. Thus, enzyme immobilization determines the greater stabilization of the enzyme structure, thanks to which the enzyme maintains high activity even after a long incubation time. High activity and stability under various process conditions of the biocatalysts, which consist of lipase immobilized enzymes on a silica support, are important because during API synthesis the process conditions are variable. Often a higher temperature is needed during the process to successfully obtain an intermediate or final product with therapeutic properties. Therefore, it is important to improve the stability of immobilized biocatalysts, so they can preserve higher catalytic properties than free biocatalysts under changing conditions.

It might also be concluded that the biocatalytic systems obtained in this study should be considered promising for resolution of racemic mixture of ketoprofen methyl ester to obtain needed (R)-enantiomer of ketoprofen.

### 3.3. Enzymatic Resolution of Racemic Ketoprofen

To verify the above-stated hypothesis, the free and immobilized fungi lipase from *Aspergillus niger* was used for enzymatic conversion to catalyze enantioselective hydrolysis of (R)-ketoprofen from (R, S)-ketoprofen ethyl ester racemic mixture (Table 4). This type of lipase is more specific towards (R)- form than (S)- and it is related to the origin of the enzyme [49]. This is reflected in the obtained data. The highest efficiency of the reaction (51.1%) was obtained for the lipase immobilized by physical immobilization by the adsorption interactions and partially interfacial activation in PBS 7 and the process was conducted for 4 days, whereas 20 mg of immobilized enzyme was used. Similar results were obtained for a lower amount of the biocatalyst, where the efficiency equaled 46.3%. Around 45% process efficiency was reached using 20 mg of lipase mixed physical immobilization via interfacial activation and ion exchange. 

These data correspond to the values of activity retention, which showed that lipase immobilized by physical immobilization by the adsorption interactions and partially interfacial activation retained higher activity than lipase mixed physical immobilization via interfacial activation and ion exchange. Interestingly, only a slight increase in process yield, as compared to 24 h of process time, was observed when the reaction duration was prolonged to 96 h. From an economical perspective, the 24 h process is more suitable for this reaction due to the fact that the efficiencies for most of the performed reactions are higher and take a shorter time. Thus, the next tests were performed for 24 h. In the pure organic solvent, efficiencies were relatively low: for lipase immobilized by physical immobilization by the adsorption interactions and partially interfacial activation, where the process was conducted for 24 h in hexane as a solvent, the efficiency was at the level of 46%. For most of the samples conducted in organic solvents, the drop in efficiency is visible, because the organic solvent interferes with the enzyme active site resulting in lower values of process efficiency. For all processes, the enantiomeric excess was very high and mostly exceeded 98% and more. As shown in the literature, the efficiencies but also enantiomeric excess is very much related to the type of enzyme used in the process. Lipases may differ from each other with the parameters like optimal temperature range, pH, or specificity in synthesis processes.

Lipase from *Candida antarctica* (CALB) has the same enantioselectivity toward (R)-ketoprofen, exactly like lipase from *Aspergillus niger*. These properties, on the other hand, can be used in an unconventional way to obtain (S)-ketoprofen. For example, Ong et al. used lipase in the esterification of the racemic mixture of ketoprofen in order to obtain a pure (S)-enantiomer [50]. They obtained enantiomeric excess of the product at the level of 66.4%. In another study, Xiang et al. used lipase from *Burkholderia cepacia* immobilized on polymeric resin for ketoprofen resolution. Due to the enantioselectivity of the enzyme toward the (R)-enantiomer, during the esterification process mainly the (R)-ketoprofen ester was obtained; however, traces of (S)-ketoprofen were also detected. Enantioselectivity varied from 1.06 to 1.91, depending on the type of alcohol used in the esterification reaction [51].

## 4. Conclusions

In the presented study, lipase was immobilized using silica nanoparticles to catalyze the process of kinetic resolution of ketoprofen methyl ester. During this research two biocatalytic systems were obtained, with the use of physical immobilization by the adsorption interactions and partially interfacial activation and mixed physical immobilization via interfacial activation and ion exchange. Lipase from *Aspergillus niger* immobilization resulted in production of highly stable and active biocatalysts that retained over 80% of their activity over a wide range of process conditions, mainly due to the protecting effect of the support material and irrespective of the immobilization approach used. The highest activity of lipase physical immobilization by the adsorption interactions and partially interfacial activation was observed at temperature of 40 °C and pH 7, whereas for the second used technique the most suitable reactions conditions were 20 °C and pH 8. These results indicate the obtainment of biocatalysts suitable for application in API synthesis due to their great stability. Taking into account the obtained results of enantiomeric excess, it is noticeable that lipase from *Aspergillus niger* has a preference over directing the reaction towards creation of (R)-ketoprofen. The efficiencies and enantiomeric excess obtained during biocatalytic reactions were at the level of around 46% and above 98%, respectively, irrespective of the type of the immobilized lipase and solvent use, suggesting that selected lipase is effective in this type of reaction and can be successfully used during production of (R)-enantiomer of ketoprofen. The highest process efficiency and enantiomeric excess were obtained for the biocatalyst obtained by physical immobilization by the adsorption interactions and partially interfacial activation, where 51.1% and 97.1% were obtained, respectively. The presented biocatalytic approach can significantly reduce the use of organic solvents by allowing reactions to be carried out using only aqueous solutions such as buffers. Furthermore, thanks to the use of immobilized enzymes, it is possible to obtain the desired product, omitting a number of additional chemical reactions and harmful reagents. The conducted research proves the advantages of immobilized lipase in practice and makes it possible to obtain the desired products in a simple way. In the future, the presented systems might be used at the stage of ketoprofen ester production in order to obtain pure (S)-enantiomer.

## Figures and Tables

**Table 1:** Enzyme characteristic before and after immobilization process.

Analyzed Parameter	Free Enzyme	Immobilization (Nonmodified)	Immobilization (Modified)
Immobilization yield (%)	-	93	86
Amount of immobilized enzyme (mg/g)	-	205	187
Specific activity (U/mg)	57	53	49
Activity recovery (%)	100	90	87

**Table 2:** Particle size distribution of the pure silica and biocatalyst with lipase immobilized by physical immobilization by the adsorption interactions and partially interfacial activation and mixed physical immobilization via interfacial activation and ion exchange on silica nanoparticles.

Type of Sample	PdI	Particle Size Range
Silica nanoparticles	0.225	59–220 nm
Immobilized lipase (nonmodified)	0.805	91–1106 nm
Immobilized lipase (modified)	0.935	1484–6439 nm

**Table 3:** Amount of the immobilized lipase by two different methods remaining after desorption process, at different conditions, after 6 h. All experiments were performed in duplicate and the results presented in each of the graphs are shown as a mean value, for which standard deviation (error value) does not exceed 5%.

Type of Eluent	Amount of Immobilized Enzyme (mg/g)	
	Nonmodified Support	Modified Support
Triton X-100	42.3	51.9
NaCl 0.1 M	29.7	32.4
Triton X-100 1% + NaCl 0.1 M	17.3	20.7
NaCl 0.5 M	8.3	10.1

**Table 4:** The efficiency of the process after enzymatic conversion of ketoprofen methyl ester and enantiomeric excess (ee%) of (R)-ketoprofen over (S)-ketoprofen after 24 h and 96 h.

Immobilization Type	Amount of Enzyme (mg)	Solvent	Time (h)	Efficiency (%)	(R)-Ketoprofen *ee* (%)
Free lipase	50	PBS 7	96	21.7	99.7
Nonmodified support	10	PBS 7	96	27.4	99.7
Nonmodified support	15	PBS 7	96	46.3	99.9
Nonmodified support	20	PBS 7	96	51.1	97.1
Modified support	10	PBS 7	96	21.7	98.6
Modified support	15	PBS 7	96	32.3	100.0
Modified support	20	PBS 7	96	44.1	100.0
Nonmodified support	10	PBS 7	24	23.3	99.5
Nonmodified support	15	PBS 7	24	38.0	99.7
Nonmodified support	20	PBS 7	24	45.4	98.8
Modified support	10	PBS 7	24	20.2	100.0
Modified support	15	PBS 7	24	29.7	99.8
Modified support	20	PBS 7	24	40.3	98.1
Nonmodified support	10	hexane	24	46.0	98.9
Modified support	10	hexane	24	6.6	95.4
Nonmodified support	10	diisopropyl ether	24	19.8	98.8
Modified support	10	diisopropyl ether	24	9.9	96.9
Nonmodified support	10	isopropanol	24	12.4	98.6
Modified support	10	isopropanol	24	6.5	100.0

## Data Availability

Not applicable.
